# Mechanistic Insights into Polyphenols’ Aggregation Inhibition of α-Synuclein and Related Peptides

**DOI:** 10.1021/acschemneuro.3c00162

**Published:** 2023-04-26

**Authors:** G. F. Martins, C. Nascimento, N. Galamba

**Affiliations:** BioISI—Biosystems and Integrative Sciences Institute, Faculty of Sciences of the University of Lisbon, C8, Campo Grande, Lisbon 1749-016, Portugal

**Keywords:** neurodegenerative diseases, protein aggregation, aggregation inhibitors, α-synuclein, hydrophobic effect, proteinopathies, synucleinopathies, polyphenols

## Abstract

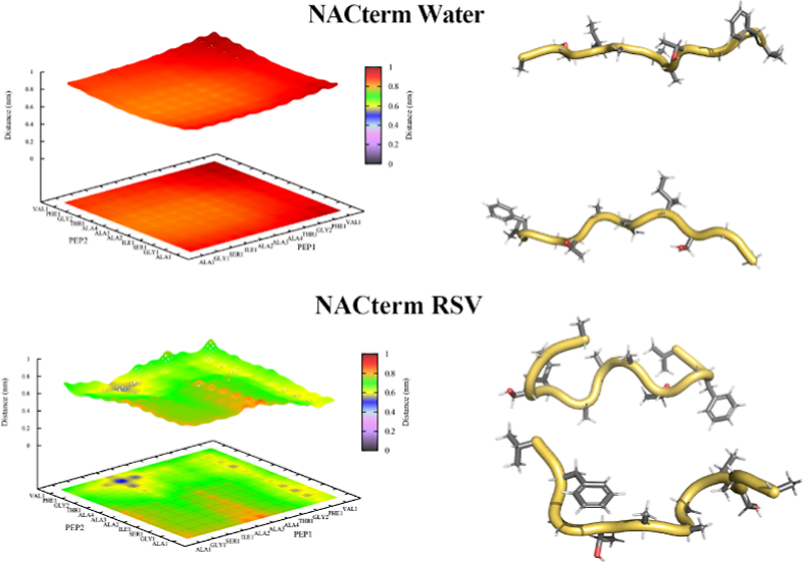

While several polyphenols were found to either inhibit or modulate the aggregation of proteins implicated in neurodegenerative diseases, such as Parkinson’s disease (PD), discrepant action mechanisms have been reported. This, in addition to some polyphenols’ pan-assay interference compounds’ reputation, casts some doubts concerning their therapeutic relevance. Here, we studied, through molecular dynamics and enhanced sampling methods, the aggregation of 11-mer peptides from the non-amyloid-β component, an aggregation-prone domain of α-synuclein (α-syn) implicated in PD and other synucleinopathies, in neat water and aqueous solutions of resveratrol (RSV) and gallic acid (GA). Further, simulations of the complete protein were carried out in aqueous urea, RSV, and GA solutions. Our results show that peptide aggregation is not disrupted by either phenolic compound. Thus, instead, intrusion of RSV and GA in the inter-peptide region induces a peptide–peptide re-orientation, favoring terminal interactions that manifest in the formation of barrierless solvent-separated configurations. Moreover, although the (poly)phenols induce a pronounced peptide dewetting at high concentrations, β-sheet-rich regions, a hallmark of α-syn aggregation, are not disrupted. Thus, our results indicate that, if anything, RSV and GA delay or modulate peptide aggregation at high concentrations via the stabilization of solvent-separated conformations as opposed to aggregation inhibition. Structural analysis of the full protein, however, shows that the (poly)phenols induce more extended conformations of α-syn, similar to urea, possibly also influencing its aggregation propensity. However, opposite to urea, the (poly)phenols reduce α-syn’s conformational space, likely due to steric effects and a slowdown of the solvent dynamics. These effects are concentration-dependent and possibly unattainable at therapeutic-relevant concentrations. These results suggest that the aggregation inhibition activity of RSV and GA in vitro should involve, instead, either the non-covalent binding to oligomeric intermediates or the stabilization of the monomer and/or oligomers through the formation of covalent bonds of the respective quinones with α-syn. In addition, the enhanced aggregation tendency of the peptides observed here could be associated with the formation of non-toxic oligomers, reported for some polyphenols.

## 1 Introduction

The etiology of neurodegenerative diseases (NDs) such as Alzheimer’s disease (AD) and Parkinson’s disease (PD) has been linked with the formation of aberrant cytotoxic protein oligomers.^1−3^ A major pathological hallmark of PD, in particular, is the formation of α-synuclein (α-syn) oligomers^4−7^ that accumulate in intracellular inclusions called Lewy bodies and Lewy neurites,^8,9^ being responsible for the loss of nigral dopaminergic neurons.^10,11^ Notwithstanding significant advances in understanding AD, PD, and other proteinopathies, and the report of various small-molecule and peptide drugs exhibiting aggregation inhibitory activity in in vitro models, treatments hampering the formation and/or stability of these oligomers remain unavailable.^12,13^

Among the panoply of small molecules and peptides found to inhibit protein aggregation, polyphenols^14−19^ are distinguished for showing activity in several proteinopathies, including PD.^20−37^ The aggregation inhibitory mechanism is, however, neither always understood nor coincident among in vitro aggregation models, and some polyphenols’ neuroprotection has been linked almost exclusively to beneficial effects on concomitant pathogenic events such as oxidative stress or defective mitochondrial function.^38,39^

**Figure 1:**
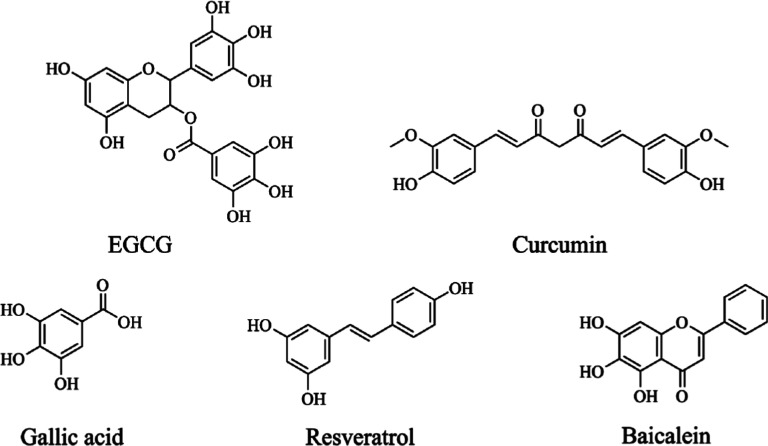
Structures of EGCG, curcumin, gallic acid, RSV, and baicalein.

Meng et al.^21^ found that the ability of flavonoids to inhibit α-syn fibrillation was associated with vicinal dihydroxyphenyl moieties irrespective of the ring position where they are located. Flavonoids with three vicinal hydroxyl groups [e.g., baicalein, epigallocatechin gallate (EGCG)—see Figure 1] exhibited enhanced aggregation inhibition on α-syn fibrillation.^21^ Aggregation inhibition was found to occur through a combined stabilization of the monomer and soluble oligomers. Furthermore, the covalent binding of the flavonoids’ respective oxidized species (i.e., quinones) to α-syn was found to be key to the α-syn fibrillation inhibition. Caruana et al.^26^ also found that the main factors underpinning α-syn self-assembly inhibition and destabilization are the existence of aromatic elements that bind to the α-syn monomer/oligomer and vicinal hydroxyl groups on a single phenyl ring; compounds with three hydroxyl groups in the same phenyl ring were found to be stronger inhibitors than those with two hydroxyl groups, with the exception of nordihydroguaiaretic acid. However, the in vitro action mechanism of polyphenols seems to depend on the experimental aggregation model. Thus, it is not clear whether the inhibition/disaggregation mechanisms in the latter study^26^ are imparted by the respective quinones through polyphenol auto-oxidation^21^ and quinone-α-syn complexation or α-syn-polyphenol non-covalent interactions, instead.

Furthermore, whereas Caruana et al.^26^ found that polyphenols such as baicalein and EGCG inhibit oligomer aggregation, other studies found out that these compounds modulate aggregation, inducing the assembly of non-cytotoxic oligomers.^27,28,40^

Ehrnhoefer et al.^27^ reported that EGCG redirects amyloid fibril formation into highly stable spherical non-cytotoxic oligomers by directly binding to the natively unfolded α-syn, preventing their conversion into β-sheet-rich structures, a key step of the nucleation stage in α-syn aggregation.^41^ Baicalein and EGCG were also shown to disaggregate preformed α-syn amyloid fibrils.^22,28^

Singh et al.^25^ showed that curcumin (see Figure 1) does not bind to monomeric α-syn but rather to oligomeric intermediates. The proposed mechanism foresees that curcumin reduces oligomer cytotoxicity by binding to hydrophobic domains, limiting water exposure and weakening hydrophobic interactions.^25^ However, curcumin did not dissociate preformed amyloids into monomeric α-syn.^25^ A combined experimental and simulation study proposed that curcumin in combination with β-cyclodextrin (β-CD) not only inhibits aggregation but also disaggregates α-syn amyloid structures in vitro.^33^ Gautam et al.^33^ hypothesized that curcumin interacted with hydrophobic and hydrophilic middle regions of α-syn, whereas β-CD interacted with aromatic residues. Similar conclusions were extended to other polyphenols in a subsequent study,^34^ with varying efficiencies, namely, resveratrol (RSV), baicalein, and EGCG (see Figure 1).

In addition to NDs and proteinopathies in general, polyphenols have long been associated with positive effects on other pathologies, including the prevention of cancer and cardiovascular disease.^42^ However, some of these potential drugs (e.g. curcumin) have also gained the reputation of pan-assay interference compounds,^43^ being identified as good drug leads in assays for different pathologies.^13^

In light of the above, it is clear that more needs to be understood concerning polyphenols’ aggregation inhibitory activity to either pursue the development of polyphenol-based drugs or settle on their inefficiency concerning this specific mechanism. For instance, Why do some polyphenols inhibit oligomer aggregation whereas others induce/modulate oligomer aggregation in different in vitro α-syn aggregation models? Can polyphenols act like protein denaturants (e.g., urea) at high enough concentrations? Why are aromatic rings and multiple hydroxyl groups important to aggregation inhibition and disaggregation? Do polyphenols enhance the solubility of α-syn? Why do some polyphenols stabilize the α-syn monomer whereas others do not seem to interact with the monomer?

To gain insight into some of these fundamental questions, we studied here the aggregation of 11-mer peptides from the so-called non-amyloid-β component^44^ (NAC) peptide, a particularly amyloidogenic region of α-syn (amino acids 61–95), through molecular dynamics simulations and enhanced sampling methods.

We probed two archetypical naturally occurring phenolic compounds, namely, gallic acid (GA; a phenolic acid) and RSV (a stilbene) (see Figure 1), both well known for their antioxidant activity, linked with multiple putative health benefits.^45,46^ The choice of RSV was motivated by the fact that this molecule is among the smallest polyphenols, whereas GA was chosen for possessing a single aromatic ring bearing three hydroxyl groups.

RSV has been reported to inhibit the aggregation of several disease-related proteins, including transthyretin, a transport protein involved in transthyretin amyloidosis,^47^ P53, a tumor suppressor protein,^48^ islet amyloid polypeptide responsible for amyloid formation in type 2 diabetes mellitus,^49^ as well as to inhibit Aβ peptide fibril formation and oligomer toxicity, although not oligomerization.^50−52^ RSV was also found to be a mild aggregation inhibitor and a poor fibril disaggregator in a dimethyl sulfoxide-induced α-syn oligomerization in vitro model.^26^ This was attributed to the small number of vicinal OH groups in the phenyl rings. However, aggregation inhibition activity was found to increase with the concentration.^26^ GA was also reported to inhibit Aβ peptide fibril formation^53^ and α-syn aggregation.^54^

While these compounds, as most polyphenols, have low solubility, absorption, and poor bioavailability, this will not concern us here as our main goal is to understand the action mechanism of simple polyphenols in α-syn-related peptide aggregation. Thus, most of our simulations are carried out at supersaturated concentrations. Nonetheless, we stress that several chemical^55^ and drug delivery strategies^56^ have long^57^ been discussed to overcome the above limitations.

## 2 Methods

### 2.1 α-syn, Peptides, and (Poly)phenols

**Figure 2:**
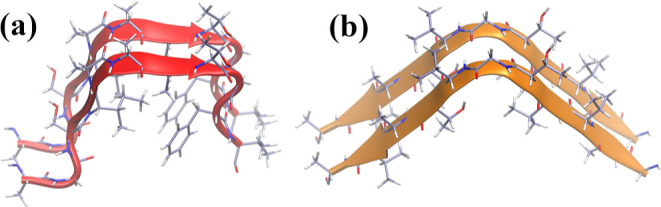
(a) NACterm “dimer”, _85_(**A**_1_G_1_S**IA**_2_**A**_3_**A**_4_TG_2_***F*V**)_95_, extracted from the α-syn experimental protofibril.^72^ (b) NACore “dimer”, _68_(G_1_**A**_1_**V**_1_**V**_2_T_1_G_2_**V**_3_T_2_**A**_2_**V**_4_**A**_3_)_78_, extracted from the α-syn experimental protofibril.^72^ Bold—hydrophobic amino acids with aliphatic side chains; Bold italic—hydrophobic amino acids with aromatic side chains. β-sheet motifs are displayed through cartoon representation—backbone hydrogen bonds are omitted for clarity.

Recently, we studied^58^ the molecular mechanism of urea concerning the aggregation inhibition of an 11-mer peptide (_85_AGSIAAATGFV_95_) from the C-terminal domain of NAC (amino acids 61–95), referred to therein as NACterm. This segment was shown to be involved in conformations of the monomer of α-syn with the potential to inhibit aggregation.^59^ NACterm (see Figure 2a) comprises the largest segment (i.e., _88_IAAA_91_) of contiguous hydrophobic amino acids and the only aromatic amino acid (Phe94) of NAC. Here, in addition to NACterm, a distinct 11-mer segment, coined^60^ NACore (_68_GAVVTGVTAVA_78_), was studied (see Figure 2b). NACore was chosen because of its relevance to the aggregation and cytotoxicity of α-syn^60^ in addition to the absence of aromatic amino acids. A slightly smaller region, encompassing residues 68–76, had been previously suggested to be pivotal to the cytotoxicity of α-syn.^61^ We note that various segments of the NAC have been associated with the aggregation and cytotoxicity of α-syn.^59−66^ In addition, N-terminal domains in the NAC flanking regions have also been associated with α-syn’s aggregation mechanism and function,^67,68^ including the domain comprising amino acids 46–53 where some missense mutations^69−71^ (e.g., A53T, E46K) associated with familial PD occur.

The peptides were studied in neat water and aqueous trans-RSV and GA either with a single RSV/GA molecule or in supersaturated solutions at 298 K and 0.1 MPa; since we are not interested in the role of the carboxyl group of GA, its protonated form was used. The solubility of RSV is extremely low^57^ (∼0.3 mg × 100 mL^–1^ = 30 mg/L = 0.13 mM). The potentials of mean force (PMFs) (aka binding free energy profiles) were computed at a (RSV/W) ratio of (15:9200) molecules, which corresponds to nearly 0.09 M; PMFs at a (1:9200) ratio were also computed; however, these showed nearly no effect, suggesting that a single molecule of RSV does not significantly perturb peptide aggregation.

Addition of more than one molecule of RSV showed that RSV aggregated, as expected, under supersaturated conditions; the PMF of distinct RSV models is discussed below. The solubility of GA in water is larger^73^ (1.47 g × 100 mL^–1^ = 14.7 g/L = 86 mM). The PMFs were computed for a (GA/W) ratio of (1:9200) and (25:9200); the latter corresponds to nearly 0.15 M, thus, again at a supersaturated concentration.

MD of the full protein of α-syn was also performed in neat water and in aqueous solutions of the (poly)phenols. The RSV and GA concentrations were 42 mM (50 RSV and 50 GA molecules). However, simulations with a single RSV molecule and at 4.2 mM (5 RSV molecules), and a simulation with 100 GA molecules (84 mM), in a similar side-length box, were also performed. In addition, α-syn was simulated in an 8 M aqueous urea solution for comparison purposes.

The use of supersaturated aqueous RSV and GA solutions should maximize the potential effect of the phenols on peptide aggregation,^26^ assuming a monotonic increasing concentration dependence. This, in addition, promotes competition between protein-(poly)phenol and (poly)phenol-(poly)phenol interactions, mimicking, to some extent, interactions with other molecules in a cell-like environment, including crowding effects since RSV and GA can form large aggregates.

### 2.2 Molecular Dynamics

Molecular dynamics (MD) simulations in the isothermal-isobaric (*N*, *p*, *T*) ensemble of NACterm and NACore in the zwitterionic form were performed in water and aqueous (poly)phenol solutions with GROMACS.^74^

The *T* and *p* were controlled with the Nosé-Hoover thermostat^75,76^ and the Parrinello–Rahman barostat,^77^ and the equations of motion were solved with the Verlet leap-frog algorithm with a 2 fs time-step. Electrostatic interactions were computed via the particle-mesh Ewald (PME) method.^78^ A cut-off of 1 nm was used for non-bonded van der Waals and for the PME real space electrostatic interactions. Heavy atom–hydrogen covalent bonds were constrained with the LINCS algorithm.^79^

The full protein systems were equilibrated for 250 ns in the *NpT* ensemble following steepest descent energy minimization and a 500 ps equilibration period in the *NVT* ensemble. The trajectories were then propagated in the *NpT* ensemble for 1 μs in water and 500 ns in the remaining systems. A relatively long equilibration period was required to observe *R*_g_ fluctuations around an average value, with the *R*_g_ systematically decreasing throughout part of the equilibration period. Thus, the starting *R*_g_ value, corresponding to the protein in a protofibril (*R*_g_ = 3.7 nm), was found to be much larger than the average values found here for the monomer. The details about the peptides’ PMF simulations are discussed below. The secondary structure^80^ of the peptides and α-syn was assessed with the program DSSP.^80,81^

### 2.3 Force Field

The peptides were simulated with the AMBER99sb^82^ force field in TIP4P-Ew^83^ model water. The general AMBER force field (GAFF)^84^ was used to build two force fields for RSV differing in the electrostatic charges. The latter were computed using the restrained electrostatic potential (RESP)^85,86^ and AM1-BCC^87^ methods for comparison purposes. The structure of the RSV molecule was optimized at the B3LYP^88^/aug-cc-pvtz theoretical level, and the Merz-Kollman^89^ charges were computed at the HF/6-31G* theoretical level. The latter calculations were performed with the program GAUSSIAN 09.^90^ A CHARMM general force field (CGenFF)^91^ was also built for RSV using CHARMM-GUI^92^ for comparison purposes. The partial charges of the distinct models are given in Figure S1 and Table S1 of the Supporting Information.

RSV and GA are solids at room temperature with melting points around 260 °C. The lack of experimental data for aqueous RSV solutions, such as hydration free energies, hampers validation of the force fields, which motivated the comparison between the different force fields commonly used to model small-molecule drugs. To probe the differences between the RSV models, we calculated the PMF of a pair of RSV molecules in water (see Figure S2) through umbrella sampling (see details below); the reaction coordinate, ξ, was selected to be the center of mass distance between the RSV molecules; the MD simulations with the CGenFF were performed with the mTIP3P^93^ water model. In spite of the differences, the distinct models predict a contact pair minimum at similar distances (GAFF/Resp: 0.37 nm; GAFF/AM1-BCC: 0.39 nm; CGenFCC: 0.42 nm) and the absence of a solvent separated pair. Although the PMFs could not be validated against experimental data, we chose to run the remaining simulations with the GAFF/AM1-BCC force field since an intermediate aggregation propensity between the GAFF/RESP and CGenFF models is observed. Nonetheless, some calculations were also performed with CHARMM36^94^ and the CGenFF for comparison purposes. GA was also described by a GAFF/AM1-BCC force field.

### 2.4 Potentials of Mean Force

The PMFs^95,96^ of NACterm and NACore dimers in neat water and aqueous RSV solutions were computed through umbrella sampling.^97−99^ The PMF of NACterm in aqueous GA solutions was also assessed. The PMF of NACore in aqueous GA solutions was not assessed since a similar result to that found with RSV and for NACterm and GA solutions was expected based on the behavior of the latter systems. The reaction coordinate, ξ, was chosen to be the distance between the COM of the middle amino acid of NACterm (i.e., Ala) and NACore (i.e., Gly), respectively. Umbrella sampling MD were carried out for the dimers in a cubic box with PBC, large enough to allow a ξ separation ∼ 3.0 nm. Whereas the peptides’ COM was initially used, comparison between the PMFs in water and aqueous (poly)phenol solutions was found to be more difficult due to the effect of the (poly)phenols on the peptides’ structure and, therefore, on the COM. The starting conformations of the peptides were obtained from the α-syn protofibril reported by Tuttle et al.^72^ (PDB code: 2n0a) from solid-state NMR spectroscopy (see Figure 2).

Following steepest descent energy minimization and a 2 ns equilibration period in the *NpT* ensemble, the peptides were pulled away with a spring constant of 5000 kJmol^–1^ nm^–2^ and a pull rate of 0.01 nmps^–1^ through steered MD to generate the initial configurations. A spacing of 0.05 nm was adopted, and the umbrella sampling MD was performed for 200–250 ns after steepest descent energy minimization, a 100 ps equilibration in the *NVT* ensemble, and 20 ns equilibration in the *NpT* ensemble. The PMFs were obtained through the weighted histogram analysis method.^100,101^ The Bayesian bootstrap method^102^ was used to estimate the PMF errors. The PMFs were corrected for the entropy^103^ by adding the factor 2*RT* ln(ξ), associated with the increasing sampling volume with the ξ increase.^104^ The PMFs were then shifted to have a zero free energy at the longest separations.

For each system, 2–3 PMFs (i.e., 2–3 replicas) were carried out starting from different initial velocities. We found that the systems could fall either in a “high” or “low” free energy contact pair basin. Whereas for some systems, increasing sampling allowed crossing from the high energy to the low energy minima, for others, the system remained in the high energy minima up to 250 ns long umbrella sampling trajectories. However, when either the high or the low energy minima in water or aqueous (poly)phenol solutions are compared, similar conclusions are found. Thus, a nearly unchanged or slightly deeper contact pair state, favoring aggregation, and the appearance of a surprisingly long range solvent-separated ensemble of conformations, favoring disaggregation, is found for the peptides in the aqueous (poly)phenol solutions.

A similar approach was used to calculate the PMF of RSV (see Figure S2). The umbrella sampling trajectories were propagated for 25 ns after an equilibration period of 100 ps in the *NVT* ensemble and 2 ns in the *NpT* ensemble.

### 2.5 Solvation Free Energies

The solvation free energies, Δ*G*_solv_, of four amino acid side chains (Ala, Val, Thr, and Phe) that comprise NACore and NACterm were computed in aqueous RSV solutions at a (RSV/W) ratio of (5:5000), which correspond to 0.05 M. These were compared with values recently reported by our group in water and 8 M aqueous urea solutions using the same force fields (i.e., AMBER99sb) and method.^58^ Δ*G*_solv_ were also computed at a (RSV/W) ratio of (1:10,000) with both AMBER99sb and CHARMM36.

The solvation free energies were calculated through “alchemical” free energy simulations,^105^ with the Bennett acceptance ratio^106^ method. Further details are available elsewhere.^58,107,108^ The side-chain analogues^109^ were built by replacing the C_α_ with an H atom with the same charge of the other H–C_β_, whereas the charge of the C_β_ was changed to neutralize the side chain analogue. The remaining force field parameters were kept unchanged. Δ*G*_Sol_ were estimated by averaging over 2–3 alchemical simulations, starting from different initial velocities.

## 3 Results and Discussion

### 3.1 Solvation Free Energies

We start our discussion by pointing out the negligible effect of RSV on the solvation free energy of the aliphatic hydrophobic (Ala, Val) and hydrophilic (Thr) amino acid side chain analogues, opposite to urea^58^ (see Table 1). Thus, RSV only changes the Δ*G*_Sol_ of Phe/toluene, slightly favoring solvation and, therefore, solubility. The latter is expected since RSV and toluene can interact through π–π stacking, similar to RSV–RSV, inhibiting the formation of hydrogen-bonds (HBs) as proton acceptors (π...HW) with water molecules. Solvation should then be entropically (and enthalpically) favored through the release of a water molecule forming an HB with the aromatic ring; nevertheless, Δ*G*_Sol_ has a large uncertainty compared with the remaining side chain analogues.

**Table 1:** Solvation Free Energies of Ala, Val, Thr, and Phe Side Chain Analogues in Water, 8 M Aqueous Urea, and Supersaturated Aqueous RSV (GAFF/AM1-BCC) Solutions at a (RSV/W) Ratio (5:5000)

aa/analogue	water Exp Δ*G*_sol_ (kJ mol^–^^1^)	water MD Δ*G*_sol_ (kJ mol^–^^1^)	RSV Δ*G*_sol_ (kJ mol^–^^1^)	urea Δ*G*_sol_ (kJ mol^–^^1^)
Ala (methane)	+8.4	+10.6 ± 0.07	+10.5 ± 0.1	+11.4 ± 0.1
Val (*n*-propane)	+8.2	+11.1 ± 0.1	+10.8 ± 0.2	+10.0 ± 0.2
Thr (ethanol)	–21.0	–17.7 ± 0.1	–17.7 ± 0.1	–18.8 ± 0.1
Phe (toluene)	–3.7	+0.8 ± 0.1	–0.03 ± 0.5	–2.9(5) ± 0.2

The respective Δ*G*_Sol_ in an 8 M aqueous urea solution are also displayed in Table 1.^58^ Urea interacts favorably with hydrophilic and hydrophobic groups, enhancing the hydration of alkanes larger than ethane.^107,110^ This, in turn, strongly favors disaggregated over aggregated peptide states, suggesting that if RSV can inhibit peptide aggregation, a different mechanism should come into play.

Although not computed for an aqueous RSV solution at a (5:5000) ratio, Δ*G*_sol_ for Ala/methane, Val/propane, and Thr/ethanol computed with the CHARMM36 force field at a (RSV/W) ratio (1:10,000) (see Table S2) show that CGenFF RSV also does not impact the solvation of these amino acid analogues; a similar result was found for GAFF/AM1-BCC at a (1:10,000) ratio.

### 3.2 Potentials of Mean Force

We now discuss the aggregation of NACterm and NACore. While aggregation inhibition of such peptides by any polyphenol does not necessarily translate into a similar behavior for α-syn or other amyloidogenic proteins, it allows the probing of whether polyphenols can inhibit aggregation by interacting with these segments of NAC.

**Figure 3:**
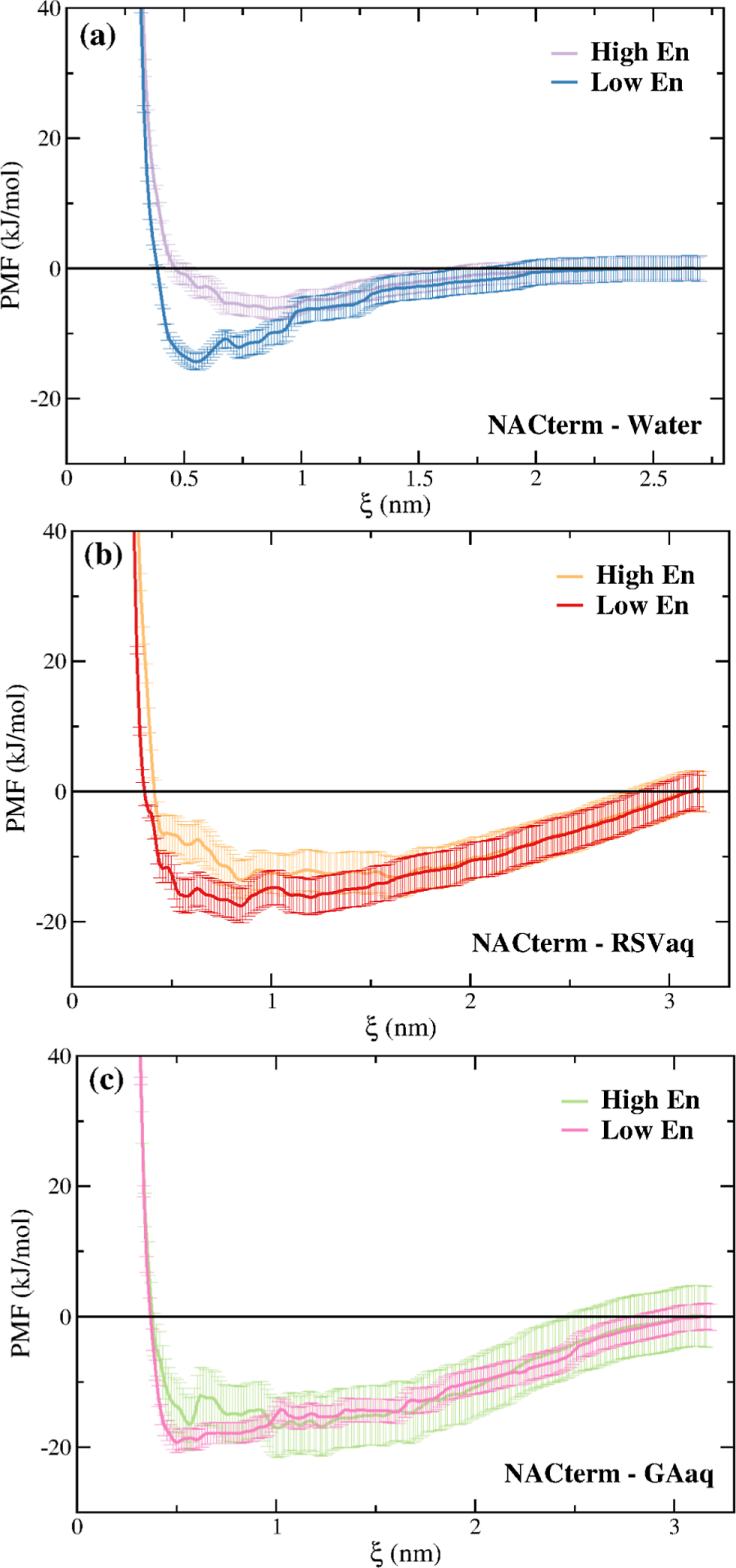
PMFs of NACterm in (a) neat water, (b) aqueous RSV, and (c) aqueous GA solutions. The replicas are distinguished in high- and low-energy PMFs.

Figure S3 displays the PMFs for NACterm in neat water and aqueous RSV and GA at a (1:9200) (poly)phenol/water ratio; no significant differences can be observed. This result motivated the use of large RSV and GA concentrations to maximize any putative effects on the free energy profiles. Figure 3a–c shows the PMFs of NACterm in neat water and supersaturated aqueous RSV and GA solutions. The PMF replicas are distinguished in “high” and “low” energy PMFs. This difference is more marked for NACterm in neat water (Figure 3a) and near the contact region, in general, with fluctuations that can be of the order of ∼2 kT to 3 kT. Block average PMFs showed that some PMFs can switch from either a high to a low energy or a low to high energy profile. For most systems, however, lower fluctuations were observed and similar block PMFs were found, suggesting convergence after ∼100 ns; running a third replica for some systems showed a profile similar to one of those obtained on the other replicas.

Nonetheless, as further discussed below, such seemingly “high” and “low” energy profiles will not influence our conclusions concerning aggregation in water and in the aqueous (poly)phenol solutions.

The most prominent feature of Figure 3 is the fact that the PMFs in aqueous (poly)phenol solutions extend through much longer distances, even precluding an accurate assessment of the zero free energy domain. The PMF, *W*(ξ), gives the free energy profile along a reaction coordinate, ξ^96,104^
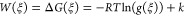
1where *g*(ξ) is the radial distribution function (RDF), that is, the probability of finding the peptides at a distance ξ, *k* is a constant, and the entropic term 2*RT* ln(ξ) was omitted. The PMF is, thus, defined up to a constant, *k*, generally set by the RDF going to 1 when ξ → ∞, that is, *G*(ξ = ∞) = 0. Here, the PMF was calculated through umbrella sampling^97−99^ to overcome sampling limitations in the calculation of *g*(ξ). However, to estimate the binding free energy, that is, the free energy difference between the peptides at an infinite separation and at the equilibrium distance, long enough distances, ξ_max_, must be sampled such that . For the aqueous RSV and GA solutions, this distance may not have been reached and the depth of the PMFs could be poorly estimated. To assess the PMF up to longer distances, a larger box was used to estimate a more accurate ξ_max_ for NACterm in an aqueous RSV solution. The discussion on the reasons behind the peculiar long distances up to which the PMF extends is postponed to the next sections. This simulation was carried out for the same 15 RSV molecules and 15,500 water molecules, reducing the concentration to 0.05 M = 50 mM, the same concentration used to compute the solvation free energies of the amino acid side chain analogues.

Figure S4 shows that whereas the PMF extends up to nearly 4.0 nm, influencing the definition of the *G*(ξ→∞) = 0, a similar qualitative behavior at short distances is observed, namely, a lower energy aggregate state (i.e., a contact pair) compared to that of neat water. Thus, if anything, a slightly deeper contact pair is observed when a larger value of ξ_max_ is used.

**Figure 4:**
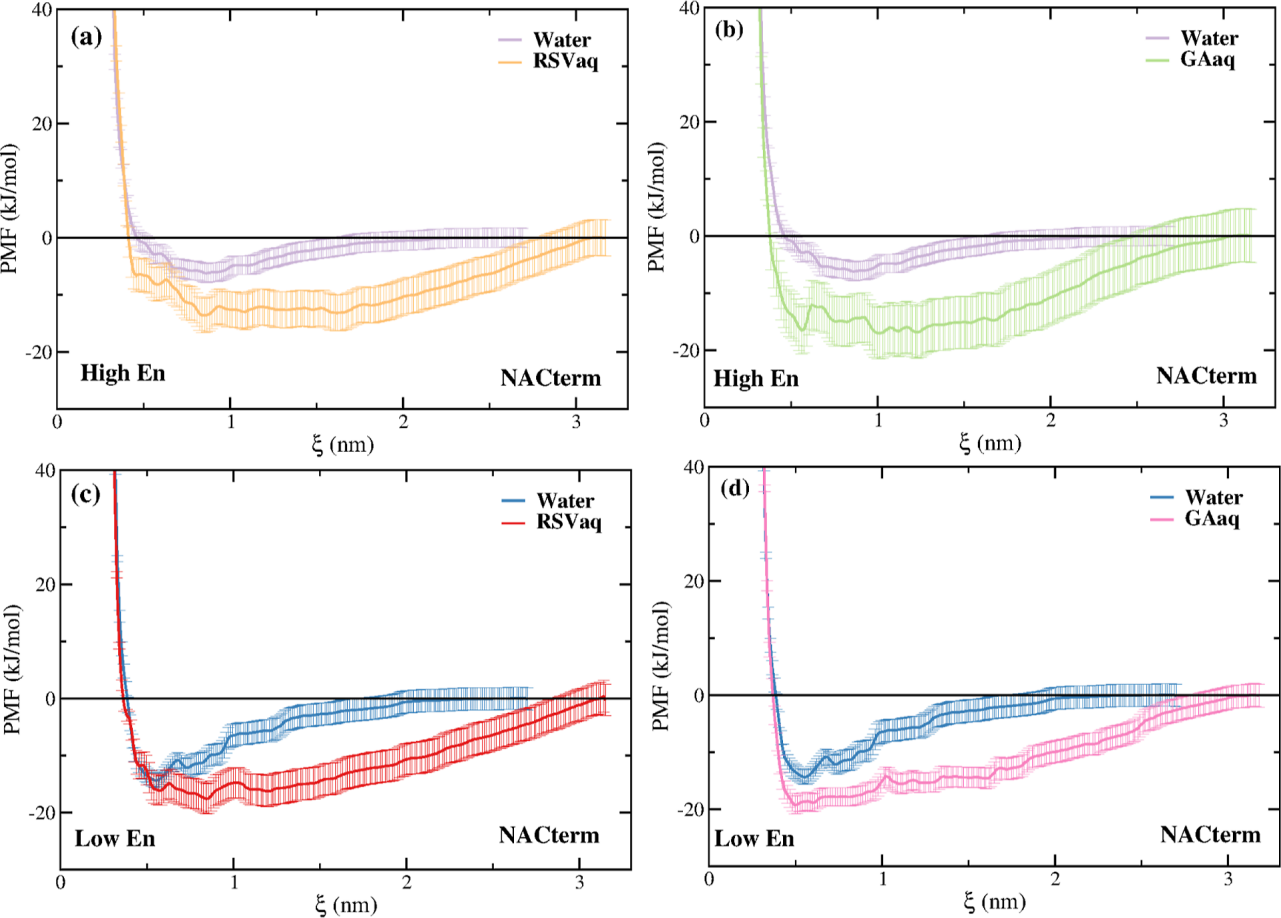
PMFs of NACterm in neat water and aqueous RSV and GA solutions.

Figure 4a–d compares the NACterm high- and low-energy PMFs, respectively, in water and aqueous RSV and GA solution. A deeper contact pair state, favoring aggregation, is found for the peptides in the aqueous RSV and GA solution. Notice that if the high- and low-energy PMFs are averaged, similar conclusions are found. We stress that this behavior was not observed for NACterm in 8 M aqueous urea solutions where a contact pair is not thermodynamically stable.^58^

**Figure 5:**
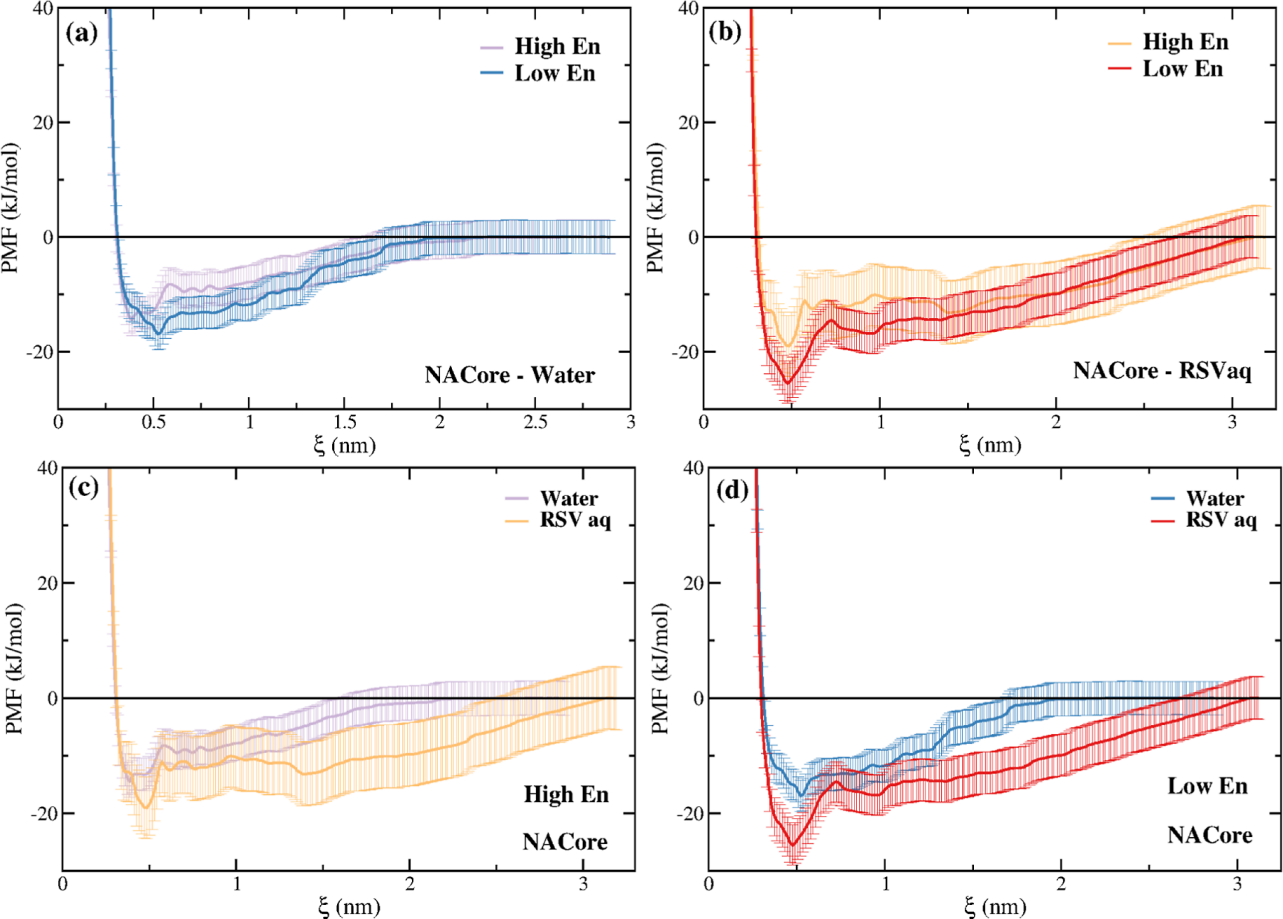
PMFs of NACore in neat water and aqueous RSV solutions.

Figure 5 shows similar plots for the NACore PMFs in water and in an aqueous RSV solution. Similar to NACterm, a deeper contact pair can be seen in the RSV solution as well as a longer tail, indicating that the PMF does not converge to zero up to ∼3 nm. When compared to NACterm, NACore is slightly more amyloidogenic in an aqueous RSV solution (see Figure S5).

To gain insight into the molecular source of the above free energy profiles, we studied the hydration, secondary structure, and the orientation of the peptides, now discussed.

### 3.3 Peptide Hydration

**Figure 6:**
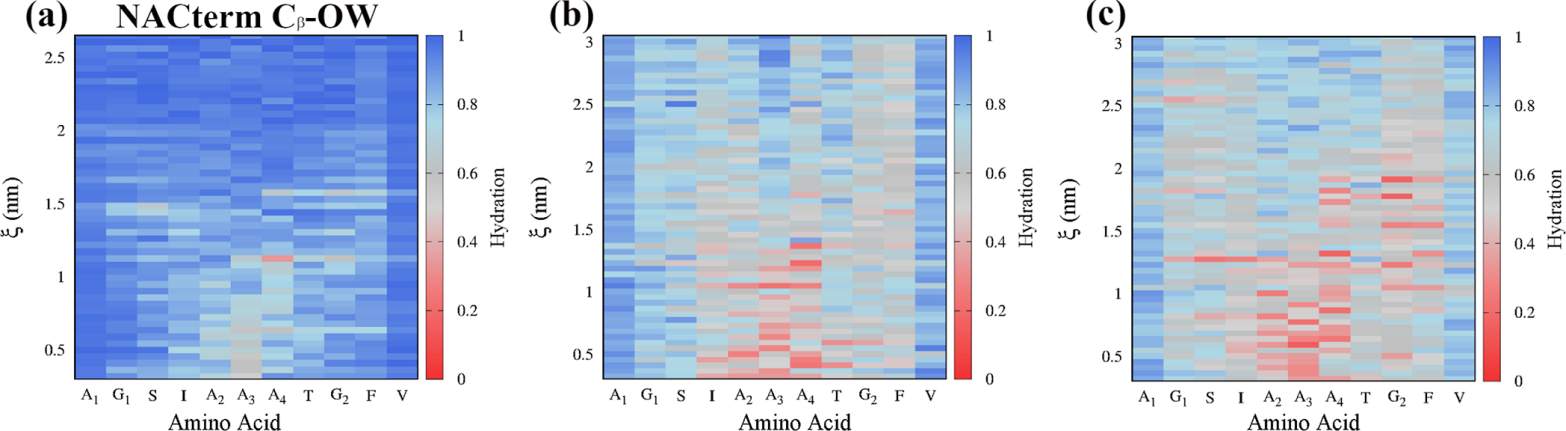
Hydration maps of NACterm amino acids (C_β_) in (a) neat water, (b) an aqueous RSV solution, and (c) an aqueous GA solution from umbrella sampling simulations.

To probe the hydration level next to the peptides, hydration maps were calculated from the umbrella sampling trajectories (Figure 6). These were built by calculating the amino acids C_β_–OW (C_α_–OW for glycine) and the backbone O–OW and N–OW (Figure S5) coordination numbers (CNs), along the PMF reaction path, where OW is the water molecules’ oxygen atom.

The CNs in neat water and in the RSV and GA solutions were all normalized by the maximum CNs for each amino acid in neat water
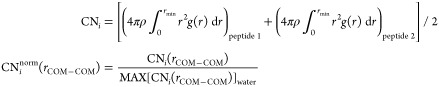
2where CN_*i*_ is the CN of amino acid *i* averaged over the two peptides, *g*(*r*) is the RDF, *r*_min_ is the first minimum of the respective RDF, and CN_*i*_^norm^ is the normalized CN for amino acid *i*.

Figure 6a–c shows a clear hydration/dehydration transition as the NACterm peptides approach. A similar dehydration is observed next to the backbone O and N atoms (see Figure S6). Notice that a more pronounced dehydration is expected in the central region, where interpeptide backbone contacts are maximized upon association, especially near A_3_, since this is used in the reaction coordinate definition. A similar result was found for NACore (see Figure S7). Figure 6 also indicates that both RSV and GA interact with the peptides, replacing water molecules, and, therefore, inducing an additional dehydration upon aggregation. Such a replacement, also observed in an 8 M aqueous urea solution,^58^ does not, however, inhibit aggregation, opposite to urea.

This means that (poly)phenols cannot suppress hydrophobic and electrostatic interactions, opposite to urea, a result consistent with the solvation free energies previously discussed.

We also calculated solvation maps based on the peptide–RSV and peptide–GA coordination. RSV and GA solvation maps (see Figure S8) show a lower intrusion of RSV than GA near NACterm and a lower intrusion of RSV near NACore than next to NACterm.

## 4 Peptides’ Relative Orientation

We now turn attention to the peculiar long-distance tail observed for the NACterm and NACore PMFs in aqueous solutions of RSV and GA. These tails indicate that the peptides still interact significantly at large values of the reaction coordinate, ξ. Such interactions must necessarily involve flanking regions of the peptides since ξ restrains any significant interaction between the central amino acid (A_3_) of the peptides. Since these long-range interactions are not observed in water, this indicates that a distinct relative orientation of the peptides should be induced by the (poly)phenols.

To assess the peptides’ relative orientation in neat water and in aqueous (poly)phenol solutions, the interpeptide amino acid–amino acid distances (*d*_aa_) were calculated at two values of the reaction coordinate, corresponding to the contact pair (ξ = 0.55 nm) and the closest distance at which the PMF in water, but not in the (poly)phenol solutions, converges to zero (ξ = 2.05 nm).

The maps were built by calculating the average distance, *d*_aa_, between the COM of each amino acid pair. The distances were then normalized by the largest *d*_aa_ (i.e., *d*_max_) among every solution (e.g., neat water, RSVaq, and GAaq for NACterm) for each peptide, *d*_aa_^*^ = *d*_aa_/*d*_max_.

**Figure 7:**
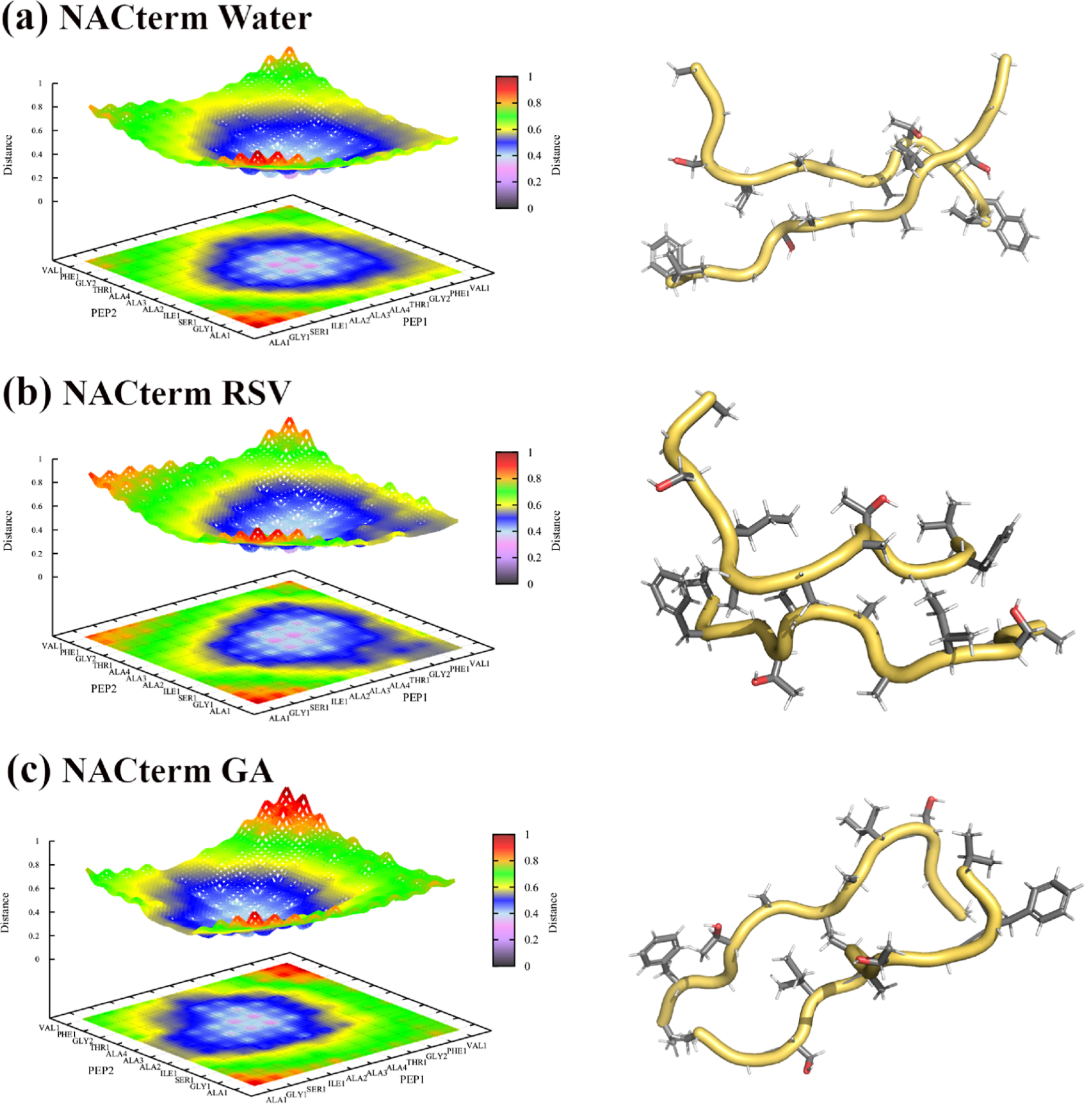
Amino acid–amino acid distance maps computed from the umbrella sampling window corresponding to the minimum (ξ = 0.5 nm) for NACterm in (a) neat water, (b) aqueous RSV solution, and (c) aqueous GA solution. The scaled reaction coordinate is ξ* = ξ/*d*_max_ = 0.26.

Figure 7 displays these maps for the NACterm in water and the aqueous solutions of RSV and GA; similar results for NACore are displayed in Figure S9. A configuration of the peptides at the respective distances is also shown in Figure 7. We note that although an antiparallel orientation was favored at ξ = 0.55 nm, observation of the trajectories at different ξ around the PMF minimum showed that the peptides can be found either in a parallel or in an antiparallel orientation.

Although some differences are perceptible in Figure 7, especially between the maps in water and in the aqueous GA solution, a similar behavior can be seen, namely, a closer distance between the central amino acids, as expected.

**Figure 8:**
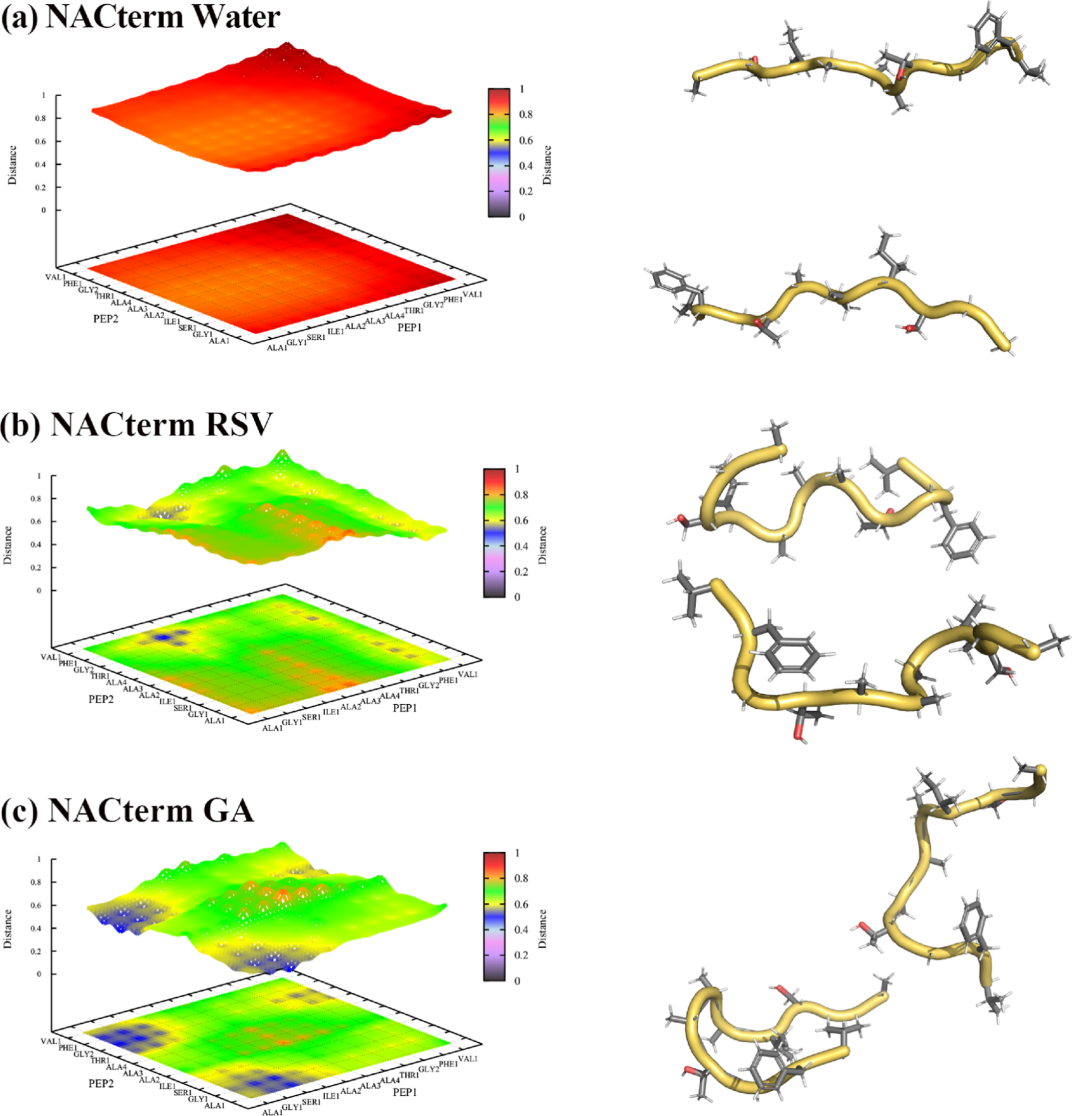
Amino acid–amino acid distance maps computed from the umbrella sampling window corresponding to ξ = 2.05 nm for NACterm in (a) neat water, (b) an aqueous RSV solution, and (c) an aqueous GA solution. The scaled reaction coordinate is ξ* = ξ/*d*_max_ = 0.81.

On the contrary, the distance maps displayed in Figure 8 for ξ = 2.05 nm show a completely different picture in water and in the (poly)phenols’ solutions.

Thus, while Figure 8a shows that the peptides are no longer in contact and reduced amino acid–amino acid distances vary between ∼0.8 and 1.0, Figure 8b,c shows that distances around ξ* = 0.81 are mainly observed in the central region because of the umbrella harmonic potential. Figure 8b shows that the Phe maintains a close contact with several amino acids of the opposite peptide. Figure 8c for GA shows an even more marked contact region between the peptides, with one of the peptides adopting a hairpin conformation. The hairpin tips are oriented toward one of the end regions of the opposite peptide giving rise to close contacts. Figures S9 and S10 show a similar behavior for NACore. Therefore, both RSV and GA seem to induce structural conformations that promote the peptides’ contact even at long values of the reaction coordinate, leading to a solvent-separated stabilization of the system not observed in neat water.

## 5 Peptides’ Secondary Structure

**Figure 9:**
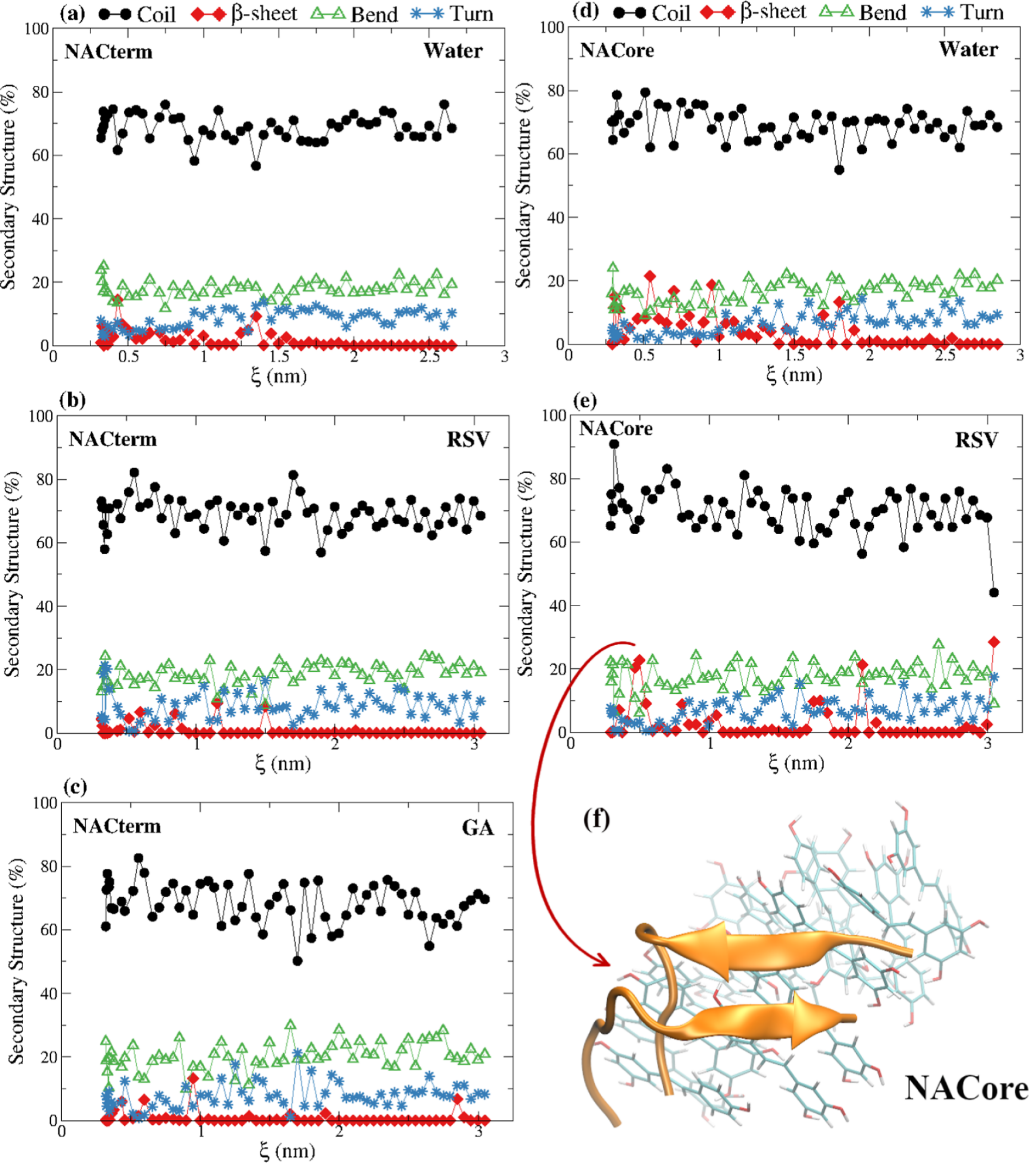
Secondary structure of the peptides calculated from the umbrella sampling trajectories in water and aqueous solutions of RSV and GA; lines are a guide to the eye.

α-syn oligomers are characterized by the formation of interpeptide β-sheet structures. In α-syn protofibrils, these structures appear in the NAC (cross β-sheets) and N-terminal domains.^72^ The secondary structure of the peptides was, therefore, also assessed here. Figure 9 shows the appearance of β-sheet structures in water, upon aggregation, especially marked for NACore.

These structures, however, persist in the aqueous solutions of RSV and GA, consistent with the PMF contact pair invariance; some prominent intra-molecular β-sheets (β-hairpin) are also observed for NACore in the aqueous RSV solution. Thus, opposite to urea,^58^ RSV and GA do not disrupt β-sheets, indicating that any aggregation inhibition mechanism is probably not directly associated with the binding to the NAC domain of α-syn.

Although our results are limited concerning the multitude of possible binding groups in the protein, RSV and GA do not display an aggregation inhibitory activity similar to that portrayed by urea, where intrusion of enough urea molecules suppresses hydrophobic interactions and backbone HBs. Notwithstanding the above results, other aggregation inhibitory mechanisms, including a possible stabilization of α-syn, cannot be ruled out, and, therefore, such a structural mechanism was also investigated.

### 5.1 α-syn Structure in Aqueous Urea and (Poly)phenol Solutions

To probe the structural perturbations induced by RSV and GA, we performed MD simulations of the full protein in water and aqueous solutions of RSV and GA. These were further compared with the structure of α-syn in an 8 M aqueous urea solution. Urea is expected to induce less compact conformations of the monomer by favoring the solvation of hydrophobic amino acids.^58,107,111^ Furthermore, since urea inhibits aggregation, resemblances among the structure of α-syn in aqueous urea and aqueous RSV and GA solutions could indicate a similar ability to reduce its aggregation propensity. The structure of α-syn was characterized by the radius of gyration, *R*_g_, and the secondary structure.

**Figure 10:**
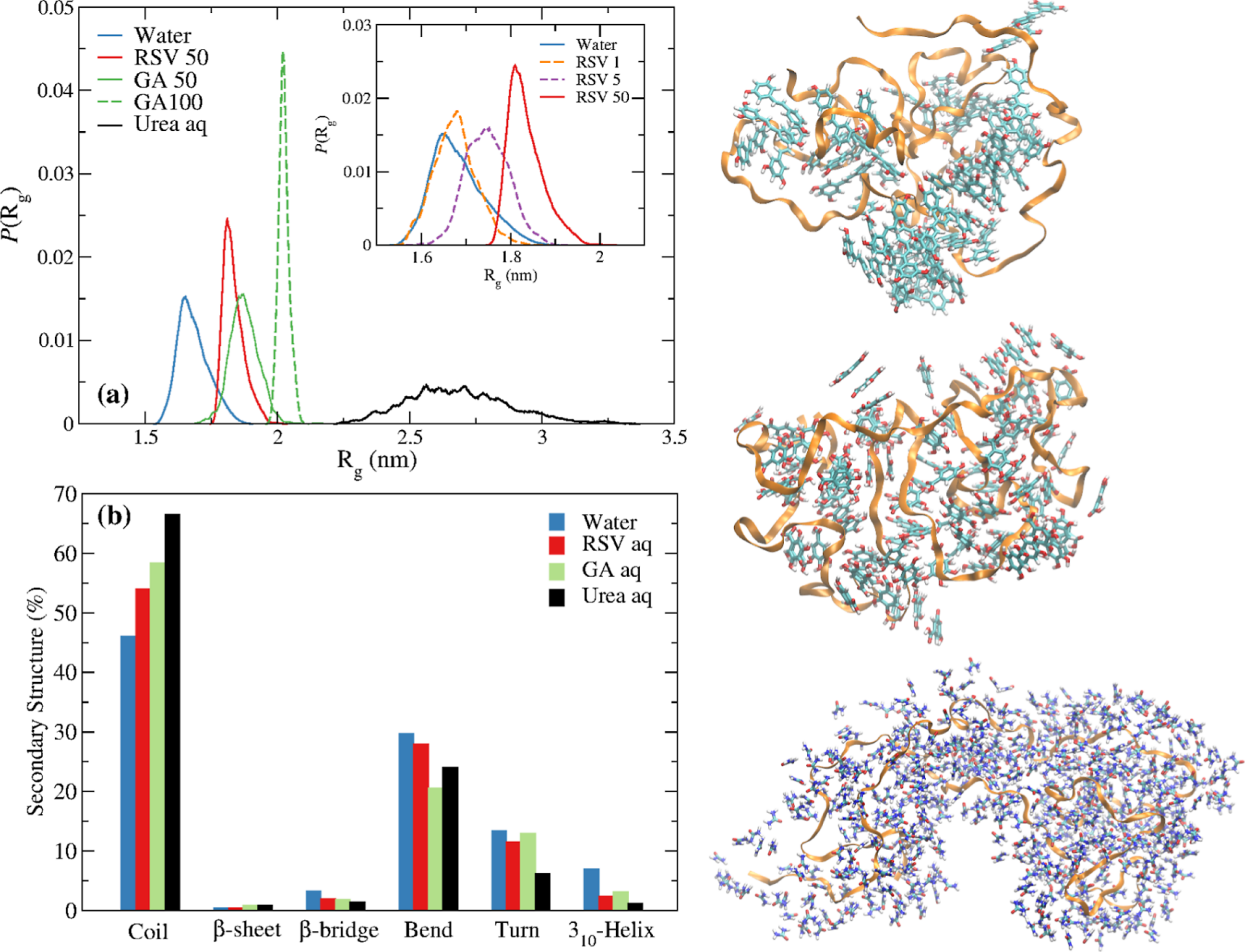
(a) Radius of gyration of α-syn in neat water, 42 mM aqueous RSV, 42 and 84 mM aqueous GA, and 8 M aqueous urea solutions. The *R*_g_ in the initial (experimental) configuration of the protofibril was *R*_g_ = 3.7 nm. The average *R*_g_ of AMBER99sb α-syn is 1.6 nm in water, 2.7 nm in urea, 1.8(1) nm in RSV, and 1.8(7) and 2.0(2) nm in GA; inset: radius of gyration of α-syn in RSV (1 RSV molecule, 5 RSV molecules, and 50 RSV molecules). (b) Secondary structure of α-syn in the different solutions; for GA, results for 50 GA are displayed; errors bars are negligible and were omitted for clarity. MD snapshots of α-syn in aqueous RSV, GA, and urea solutions are displayed on the right-hand side: RSV, GA, and urea molecules within 0.5 nm of the protein, respectively, are displayed; solvation water molecules are omitted for clarity.

Figure 10a shows the distribution of *R*_g_ for α-syn in the distinct solutions. The average value of *R*_g_ in neat water is 1.6 nm. This is much lower than the experimental values (2.5–4.0 nm) inferred from different experimental techniques;^41,112−115^ the *R*_g_ of the starting configuration of α-syn in the protofibril is 3.7 nm. All-atom force fields such as AMBER and CHARMM are known to underpredict the *R*_g_ of IDPs in general, including α-syn, portraying a more compact, *albeit*, still disordered protein.^116^ The reason is that these force fields overestimate protein–protein and/or underestimate protein–water interactions since they were primarily developed to describe the structure of globular proteins. We note that less compact structures have been reported with some optimized coarse-grained models^117,118^ and modified water models.^116^ Nonetheless, despite the AMBER99sb limitations in reproducing the experimental *R*_g_, we assume that the force field can qualitatively reproduce the structural transformations of α-syn in aqueous urea and (poly)phenol solutions.

Figure 10a shows that RSV and GA induce a less compact structure of α-syn in average, although more compact than that found in aqueous urea.

Interestingly, RSV and GA tend to narrow the *R*_g_ values accessible to the protein, nearly “freezing” the structure of α-syn, albeit only at a very high concentration. This is especially marked for aqueous GA at the highest concentration studied, with the *R*_g_ distribution approaching a delta function. This indicates that RSV and GA reduce the conformational space accessible to the protein by layering around the protein. This behavior may be associated with steric effects induced by the (poly)phenols next to the protein and/or a slowdown of the water dynamics around the protein. This is opposite to the behavior found in aqueous urea, where a broader *R*_g_ distribution is observed. Along with a smaller size, urea is known not to significantly disturb the water structure and dynamics.^119,120^ Our results indicate, in addition, that although the protein extends, urea still allows it to access many different conformations, opposite to RSV and GA.

The structural transformations of Figure 10a are accompanied by an increase of the random coil percentage, well correlated with the magnitude of the *R*_g_ shift (as expected) (Figure 10b). Thus, assuming that a less compact structure, as found in aqueous urea, disfavors aggregation, our results suggest that RSV and GA destabilize more aggregation-prone structures of the monomer. The magnitude of the shift is, however, relatively small at low concentrations (see Figure 10a—inset), suggesting that these molecules may not have an effective therapeutic potential via this aggregation inhibition mechanism. On the other hand, the effect of the suppression of α-syn’s conformational space on the aggregation tendency is unknown.

## 6 Conclusions

A panoply of polyphenols has been reported to either inhibit or modulate the aggregation of proteins associated with various proteinopathies, including α-syn, a key protein in PD, dementia with Lewy bodies, and multiple system atrophy, jointly known as synucleinopathies or Lewy body diseases. In addition, some of these polyphenols disaggregate preformed fibrils in vitro. This has granted several polyphenols, including many dietary flavonoids, the reputation of aggregation inhibitors. Furthermore, since some of these compounds showed the ability to disaggregate oligomers and mature fibrils, they could represent a pathway not only to prevent but also to reverse the disease, a pivotal issue, since NDs are often diagnosed late. The reported aggregation inhibition/modulation mechanisms, among different in vitro studies, can, however, be significantly different, and discrepant mechanisms have been put forward. The exact role of the aromatic rings and the importance of multiple hydroxyl groups in a single ring are not well-understood. Even the putative effect of these polyphenols on the solubility of the monomer, oligomers (soluble), and fibers (insoluble) remains insufficiently studied.

Herein, we studied the aggregation of two 11-mer amphiphilic segments (NACterm and NACore peptides) from NAC, a key aggregation-prone domain of α-syn, in neat water and in aqueous solutions of RSV and GA. Furthermore, the structure of the full protein was studied in aqueous solutions of the polyphenols and urea.

Our results indicate that peptide aggregation is not disrupted by either phenolic compound, even at exceedingly large concentrations, despite the polyphenols’ interaction with the protein, as opposed to remaining “trapped” in the water. Thus, instead, RSV and GA induce a peptide–peptide re-orientation, favoring terminal interactions that manifest in the formation of barrierless solvent-separated configurations extending over exceedingly large distances. Furthermore, the (poly)phenols do not disrupt β-sheet-rich regions, opposite to urea, which inhibits aggregation and disaggregates α-syn oligomers. Structural analysis of the full protein shows that although to a lower extent than urea, the (poly)phenols stabilize less compact structures of α-syn, thus possibly influencing its aggregation propensity. This stabilization is concentration-dependent and is accompanied by a reduction of the protein’s conformational space possibly due to steric effects and a slowdown of biological water, that is, water molecules sharing the protein’s surface with the (poly)phenols. These structural effects, nevertheless, seem unattainable at therapeutic relevant concentrations.

We stress that many alternative aggregation inhibition mechanisms, not assessed here, are possible. These include the aggregation inhibition via covalent binding of the respective quinones to the protein, the aggregation modulation by interacting with nascent oligomers, or the aggregation inhibition via the interaction with other amyloidogenic regions of the α-syn. Furthermore, some polyphenols have been reported to induce the formation of non-toxic oligomers. However, concerning the question on the aggregation inhibitory activity of RSV and GA of important NAC domains such as NACore and NACterm, shown to be pivotal to the aggregation process, our results indicate that these (poly)phenols do not inhibit aggregation.

## Supporting Information Available

Molecule of trans-RSV; atomic charges for different force fields of trans-RSV; potential of mean force for three force fields of RSV; solvation free energies of CHARMM36 Ala, Val, and Thr in mTIP3P water and RSV solutions; PMFs of NACterm in water and aqueous (1:9200) RSV and (1: 9200) GA solutions; PMF of NACterm in an aqueous RSV solution computed in a large box up to ∼3.75 nm; PMFs of NACterm and NACore in water and an aqueous RSV solution; hydration maps of the NACterm backbone in water and aqueous RSV solutions; hydration maps of NACore amino acids (C_β_) in water and aqueous RSV solution; NACterm and NACore, RSV and GA solvation maps; NACore aa–aa distance surfaces in water and aqueous RSV solution (ξ* = 0.21); and NACore aa–aa distance surfaces in water and aqueous RSV solution (ξ* = 0.82) (PDF)
