# Supplementary material for: Mechanistic Insights into Polyphenols’ Aggregation Inhibition of α-Synuclein and Related Peptides
Source: ACS Chem Neurosci. 2023 Apr 26;14(10):1905–20. doi: 10.1021/acschemneuro.3c00162 (PMC10197135; doi:10.1021/acschemneuro.3c00162)
Supplement: Supplementary file 1 — cn3c00162_si_001.pdf [file cn3c00162_si_001.pdf]

Mechanistic Insights into Polyphenols' Aggregation Inhibition of α-Synuclein and Related Peptides

Supporting Information

Martins, G. [a], Nascimento, C. [a], Galamba, N.[a,*]

[a] *BioISI – Biosystems and Integrative Sciences Institute, Faculty of Sciences of the University of Lisbon, C8, Campo Grande, 1749-016 Lisbon, Portugal.*

[*] Corresponding author. Electronic mail: njgalamba@fc.ul.pt

Force Fields

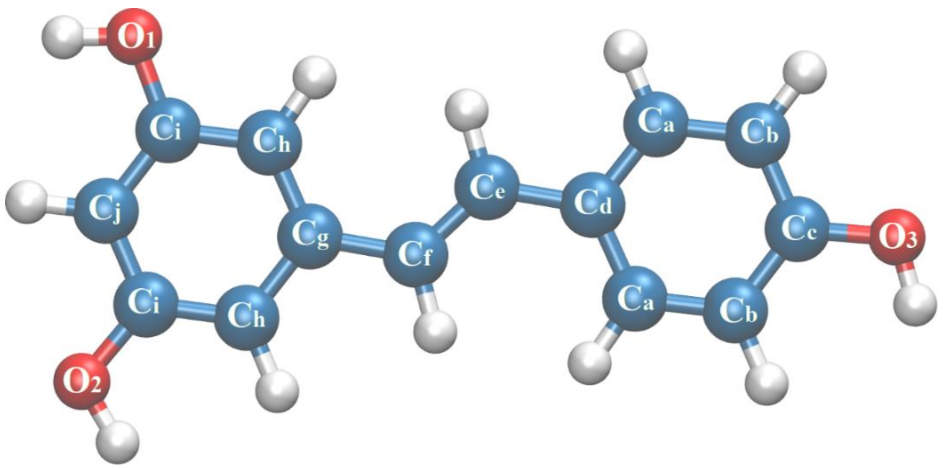

Figure S1 – Molecule of *trans*-resveratrol. The atomic charges of GAFF/RESP, GAFF/AM1-BCC, and CGenFF are given in **Table S1**.

Table S1 – Atomic charges for trans-resveratrol for the different force fields compared in this study.

Atom	GAFF/RESP	GAFF/ AM1-BCC	CGenFF
Ca	-0.0859	-0.0795	-0.117
Cb	-0.3455	-0.1865	-0.113
Cc	0.4117	0.1311	0.107
Cd	-0.0197	-0.0968	-0.033
Ce	-0.1363	-0.0852	-0.093
Cf	-0.1437	-0.1202	-0.093
Cg	0.1527	0.0182	-0.031
Ch	-0.4382	-0.2125	-0.116
Ci	0.4963	0.1601	0.108
Cj	-0.5661	-0.244	-0.111
O_1	-0.5853	-0.4951	-0.533
O_2	-0.5853	-0.4951	-0.533
O_3	-0.5362	-0.4951	-0.533
HCa	0.1365	0.1355	0.115
HCb	0.1924	0.1435	0.115
HCe	0.1638	0.125	0.132
HCf	0.1314	0.131	0.132
HCh	0.1862	0.144	0.115
HCj	0.2386	0.153	0.115
HO_1	0.409	0.4215	0.42
HO_2	0.409	0.4215	0.42
HO_3	0.3723	0.421	0.42

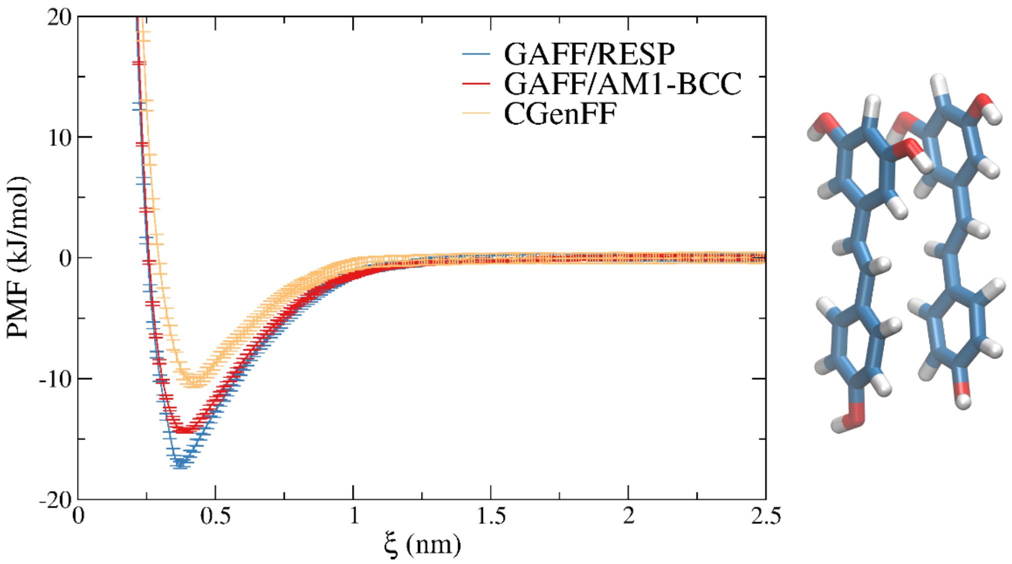

Figure S2 – Potentials of mean force in water at 298 K and 0.1 MPa for three models of RSV computed through umbrella sampling. GAFF/AM1-BCC was used to assess the PMFs of NACterm and NACore.

Solvation Free Energies

Table S2 – Solvation free energies of CHARMM36 Ala, Val, and Thr side chains in mTIP3P water and CGenFF RSV solutions at the (RSV:W) ratio (1:10000).

aa/analog	Water Exp ΔG_{sol} (kJ mol^{-1})	Water MD ΔG_{sol} (kJ mol^{-1})	RSV ΔG_{sol} (kJ mol^{-1})
Ala (Methane)	+8.4	+9.2±0.06	+9.1±0.07
Val (*n*-Propane)	+8.2	+11.2±0.1	+11.2±0.06
Thr (Ethanol)	-21.0	-21.2±0.09	-21.3±0.06

Potentials of mean Force

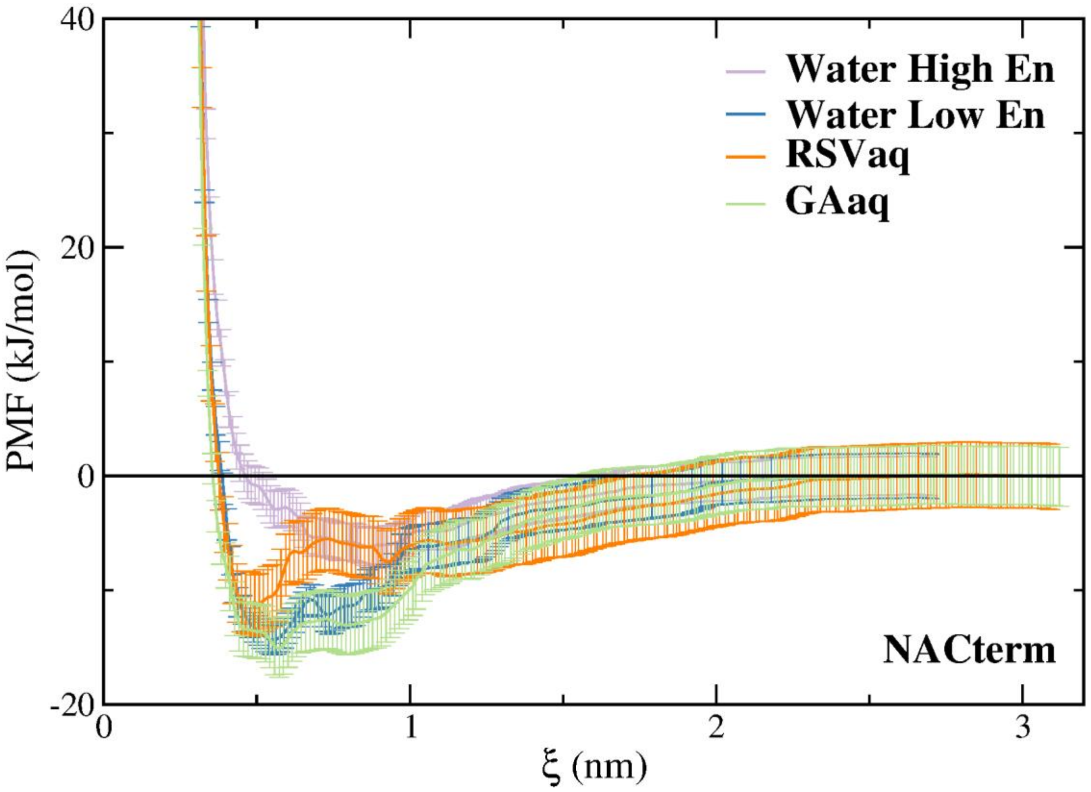

Figure S3 – PMFs of NACterm in water and aqueous (1:9200) RSV and (1: 9200) GA solutions.

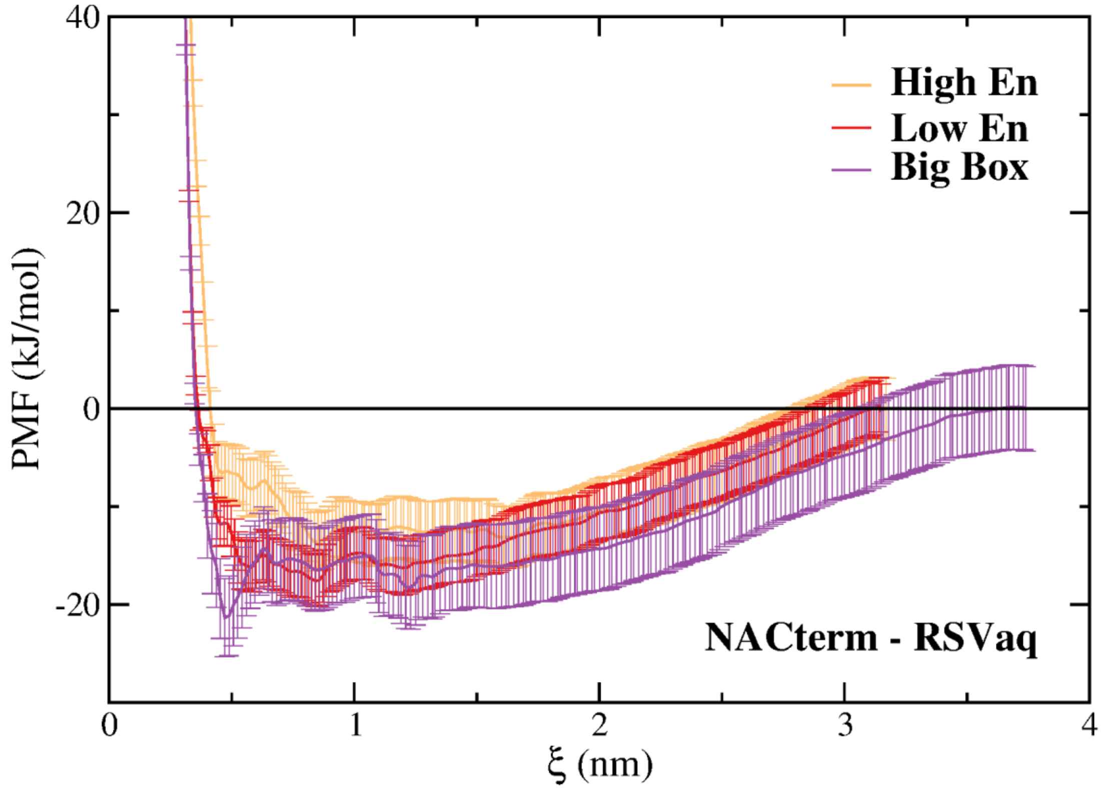

Figure S4 – PMF of NACterm in an aqueous RSV solution computed in a large box up to ~3.75 nm. The PMF is compared with that for the smaller boxes used throughout this study.

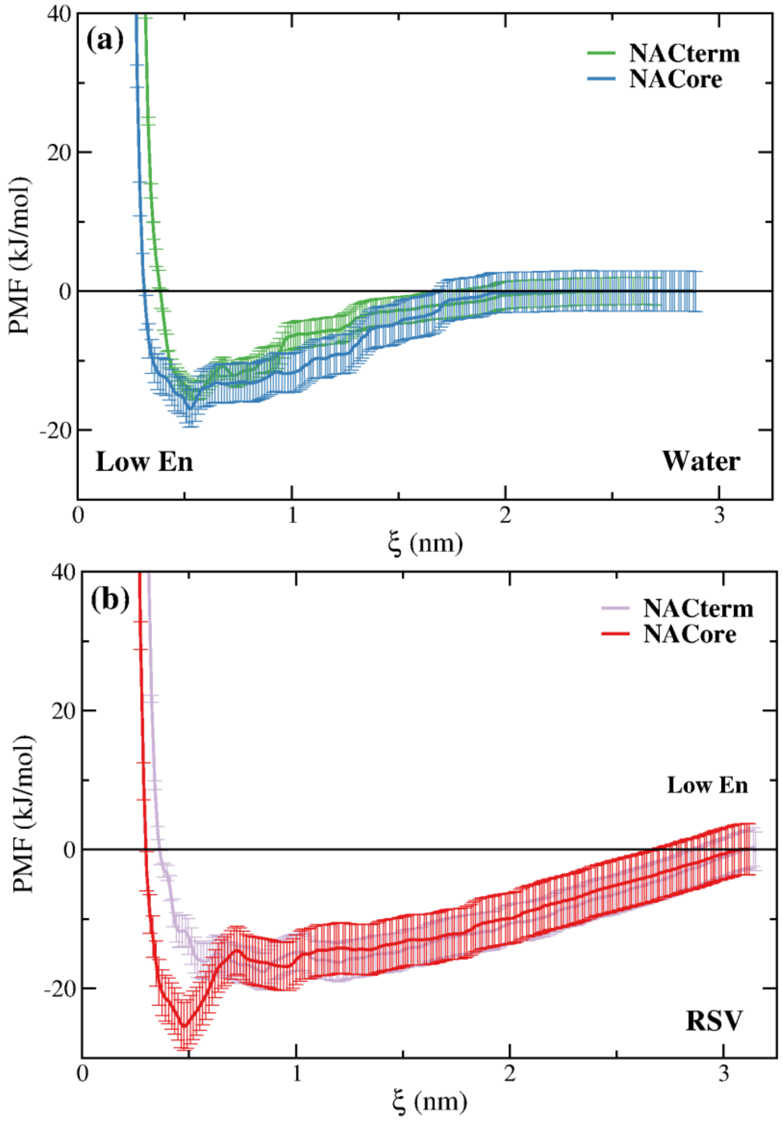

Figure S5 – PMFs of NACterm and NACore in (a) water and (b) an aqueous RSV solution.

Solvation Maps

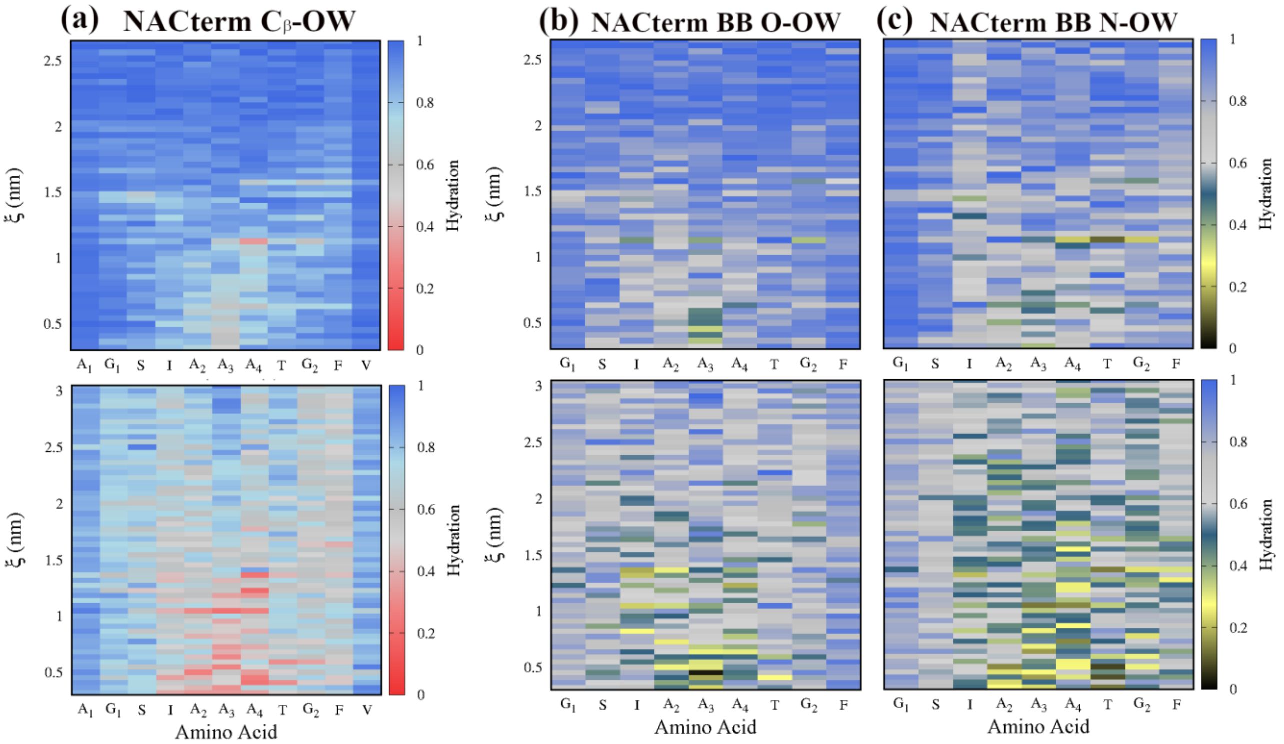

Figure S6 - Hydration maps of NACterm amino acids (C_β and backbone (BB) oxygen and nitrogen atoms) in neat water (upper) and aqueous RSV solutions (lower) computed from umbrella sampling simulations. Hydration is defined by the number of water molecules in the first hydration sphere of the C_β (C_α for Gly) and backbone O (BB O-OW) and N (BB N-OW) of the amino acids 1-11 and 2-10, respectively. Hydration in water and in the aqueous RSV solutions were normalized by the respective maximum hydration number of each amino acid in neat water (see eq. 2).

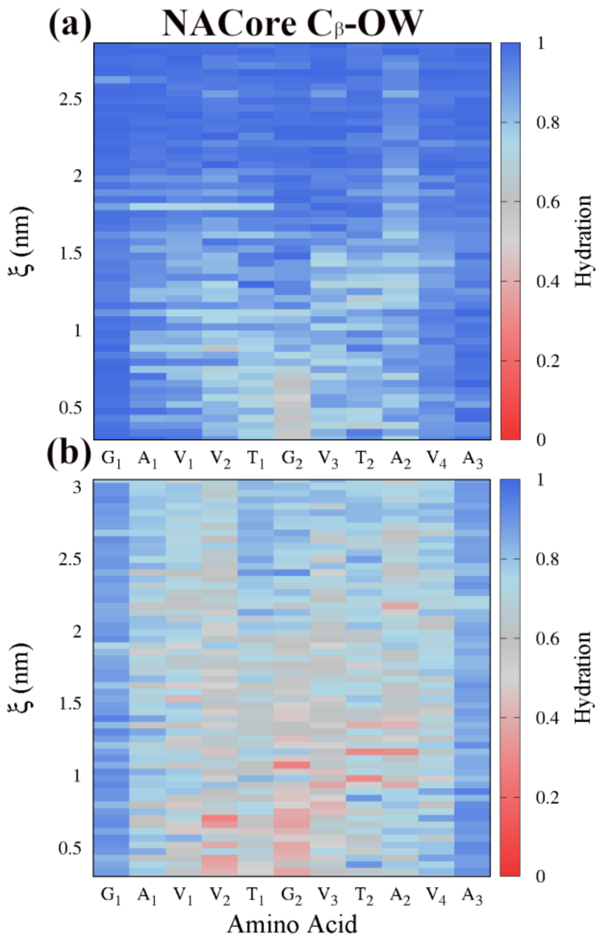

Figure S7 – Hydration maps of NACore amino acids (C_β) in (**a**) neat water and (**b**) an aqueous RSV solution, from umbrella sampling simulations.

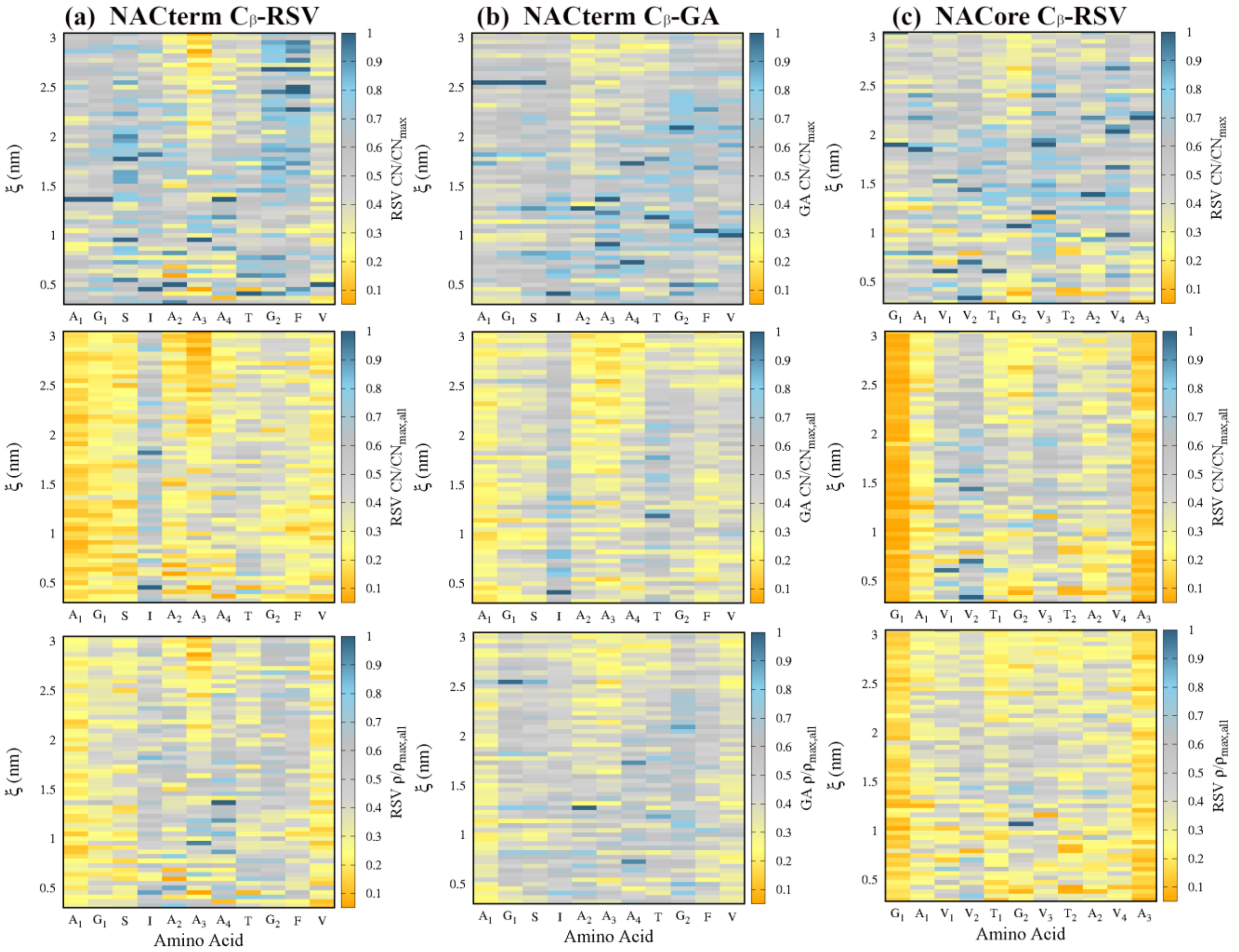

Figure S8 – NACterm and NACore, RSV and GA solvation maps computed by assessing the coordination number (CN) of the C_β of each amino acid and every heavy atom of RSV or GA, up to the aa-OW rdfs minima. (upper) the CNs were normalized by the respective maximum CN of each amino acid; (middle) the CNs were normalized by the maximum CN observed among every amino acid; (lower) the density, computed as $\rho = 4/3\pi(r_{aa,min})^3$ where $r_{aa,min}$ is the aa-OW rdfs minimum, normalized by the maximum density observed among every amino acid. Notice these maps probe the intrusion of RSV and GA within the peptides' first hydration layer as defined by the C_β-OW rdfs first minima. Phe in NACterm suffers the largest loss of RSV upon aggregation (upper panel); Ile in NACterm and Val_2 in NACore have the largest (absolute) coordination numbers (see middle panel); NACore has a lower RSV density than NACterm (see lower panel).

Peptides' Relative Orientation

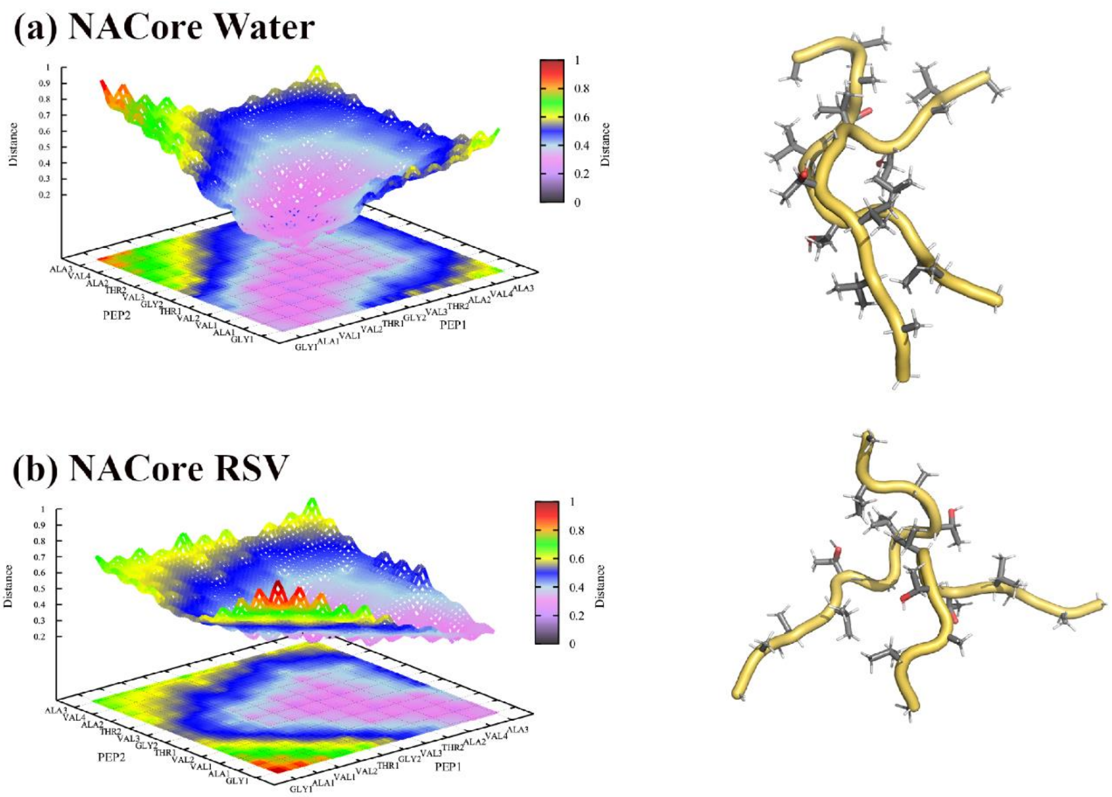

Figure S9 – NACore amino acid-amino acid distance surfaces in **(a)** water and **(b)** an aqueous RSV solution computed for an umbrella at ξ = 0.55 nm. The scaled reaction coordinate is $\xi^*=\xi/d_{max}$ = 0.21.

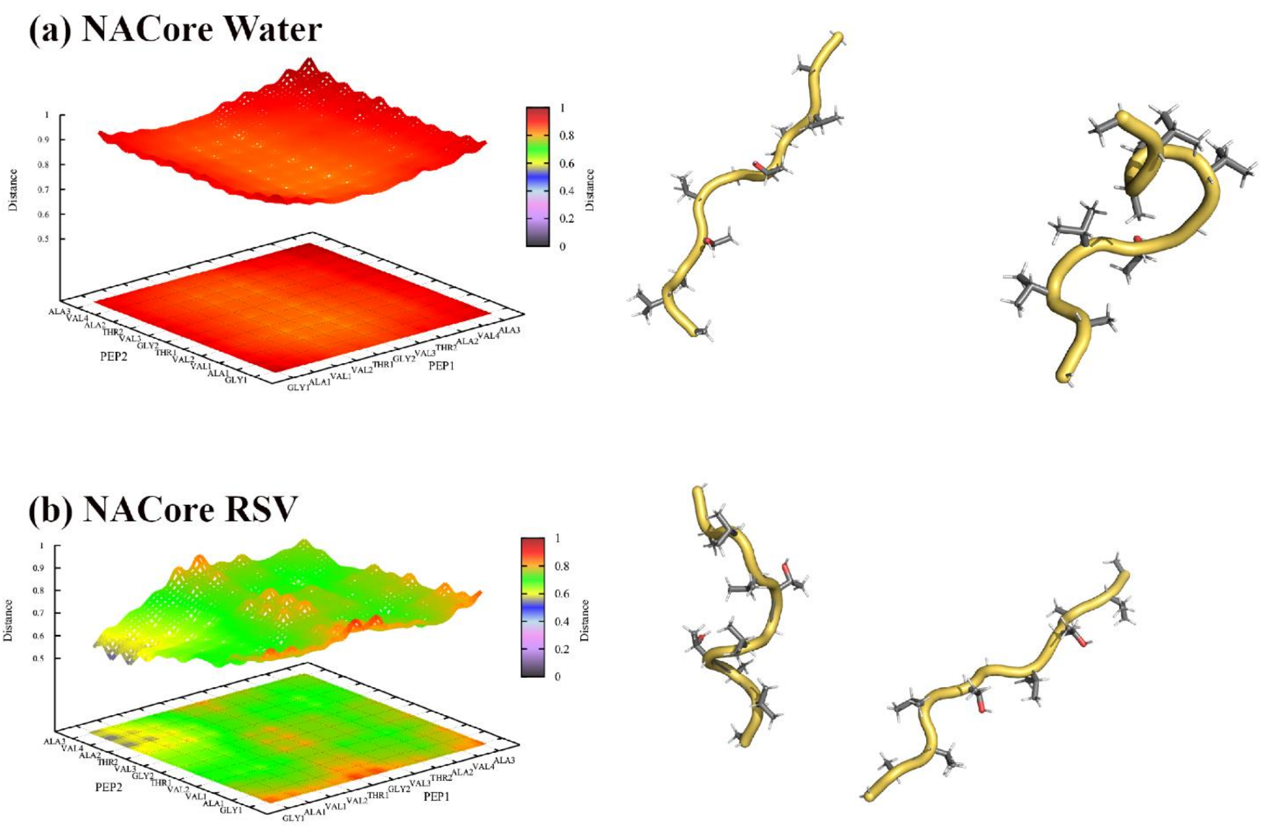

Figure S10 – NACore amino acid-amino acid distance surfaces in **(a)** water and **(b)** an aqueous RSV solution computed for an umbrella at ξ = 2.05 nm. The scaled reaction coordinate is $\xi^{*}=\xi/d_{max}$ = 0.82.
